# Comprehensive grain size database of sediments of different types of dunes in Gurbantunggut Desert

**DOI:** 10.1038/s41597-025-06242-8

**Published:** 2025-12-10

**Authors:** Zhengyao Liu, Zhibao Dong, Nan Xiao, Xiaokang Liu

**Affiliations:** 1 School of Geography and Tourism, Shaanxi Normal University, Xi’an, 710119 China; 2 Shaanxi Institute of Geo-Environment Monitoring, Shaanxi Institute of Geological Survey, Xi’an, 710054 China; 3 College of Geographical Science, Inner Mongolia Normal University, Hohhot, 010022 China

**Keywords:** Geomorphology, Environmental sciences

## Abstract

The Gurbantunggut Desert, located in the Junggar Basin of Central Asia, is China’s second-largest desert and the only mid-latitude desert dominated by extensive fixed and semi-fixed dunes. Despite its distinctive dendritic, honeycomb, and complex dune morphologies, comprehensive sedimentological datasets spanning multiple dune types and spatial positions remain scarce. Here we present a large-scale, systematic dataset of sediment grain-size distributions collected from 67 sampling zones and 424 sites, totaling 1,859 samples across diverse geomorphological units in the Gurbantunggut Desert. Surface and profile samples were collected following standardized protocols and, after rigorous pretreatment, were analyzed using a Mastersizer 2000 laser-diffraction particle-size analyzer. The dataset captures spatial variations in sediment composition among dune types, slope positions, and profiles, reflecting interactions among wind dynamics, sediment supply, and vegetation cover. This open-access dataset provides essential baseline information for studies of dune morphodynamics, aeolian sediment transport, and ecological responses in arid environments, thereby facilitating future research on source-to-sink dynamics, climate-change impacts, and desertification monitoring.

## Background & Summary

Aeolian sand landforms are important terrestrial landforms on planets and moons^1–3^. On Earth, approximately one-fifth of the land surface is covered by aeolian sediments, including inland aeolian sand landforms in arid basin zones and coastal aeolian sand landforms in coastal regions. Inland aeolian sand landforms are predominantly distributed in two latitudinal belts: between 15° and 35° latitude in both hemispheres, such as the Sahara Desert in Africa^4^ and the Simpson Desert in Oceania^5^; and between 35° and 50°N in the Northern Hemisphere, exemplified by the Karakum Desert^6^ and the Gurbantunggut Desert^7^. These inland aeolian landforms result from the interplay of arid climatic conditions and detrital materials from surrounding mountains in fault basins, while also exerting profound impacts on global human activities^8^. Moreover, climate change and intensive human activities can accelerate sand surface mobilization, leading to ecosystem degradation and affecting the global environment^9^.

Within the vast sand seas, a variety of dune forms emerge as the result of interactions between wind dynamics and surface sand sediments under arid climatic controls. Their formation and development are continuously shaped by multiple factors, including topography, sand supply, water availability, and vegetation cover^10^. The morphological characteristics, formation and evolution processes, and spatial distribution of sand dunes are central topics in aeolian geomorphology. Classic dune types such as barchan dunes^11^ and linear dunes^12^ have been well described, while more complex forms such as star dunes^13^ and parabolic dunes^14^ have gained increasing research attention. Several classification schemes have been proposed^15^, which integrate various dune morphologies into simplified categories to facilitate rapid identification, counting, and environmental interpretation. These taxonomic frameworks are fundamental to understanding the spatiotemporal evolution of aeolian sand landforms.

Sediment properties form the material basis for dune development, reflecting both depositional and erosional processes. Studies of dune sediments typically consider color, grain size, surface textures, chemical composition, and mineralogical characteristics. Among these, grain size is the most direct indicator, serving as a key parameter for reconstructing sedimentary environments. Over time, analytical methods have evolved from manual sieving to advanced approaches such as laser diffraction and the use of various sediment classification diagrams (e.g., Krumbein scale, Folk’s triangular diagrams, and Passega’s C–M plot)^16–18^. In dune systems, grain size parameters enable quantitative analyses of wind energy, sediment sorting, and geomorphological stability, and play a pivotal role in interpreting aeolian processes. Furthermore, grain size exerts critical influence on dune spacing and scale^19,20^, with important implications for studies of climate change, sediment source tracing, environmental reconstruction, and geomorphic evolution^21–23^.

The Gurbantunggut Desert is the largest fixed to semi-fixed desert in Central Asia and has attracted considerable scientific attention in geography, botany, and geology due to its unique dendritic and honeycomb dune forms, high plant diversity, and abundant oil and gas resources^24–27^. With respect to sediments, Wang *et al*. and Ha *et al*. revealed spatial differentiation in surface sediment structures from south to north^28^, indicating regional wind dynamic gradients. Ji *et al*. found that bare sand and gravel are mainly distributed in the middle to upper parts and dune crests, while other areas are covered by vegetation. Although these dunes are classified as fixed to semi-fixed, the uppermost 1–3 cm of sediment remains loose and highly susceptible to disturbance^24^. Wei *et al*. highlighted the role of grain size in regulating dune water and heat dynamics, thereby influencing vegetation growth^29^. Micromorphological and geochemical analyses of quartz grains by Qian *et al*.^30^ indicate that desert sediments derive mainly from clastic inputs from surrounding mountains and from weathering products of basin bedrock, recording combined aeolian and fluvial deposition from the late Middle Pleistocene to the late Holocene.

Despite these achievements, most prior studies in the Gurbantunggut Desert focused on localized areas or single dune types and have not comprehensively integrated grain size surveys across multiple dune types, positions, and depths at the desert-wide scale. To address this gap, this study targeted the key geomorphological features of various dune types and systematically collected surface sediment samples based on the modern geomorphological landscape of the Gurbantunggut Desert. The analyses aimed to reveal regional differences in sediment properties, depositional processes, and environmental conditions at a macroscale. On this basis, a comprehensive, open-access grain size dataset has been developed. This dataset provides fundamental data support for future research and facilitates exploration of key scientific questions regarding source–dynamic–environment interactions across different spatial and temporal scales.

## Study area

**Fig. 1:**
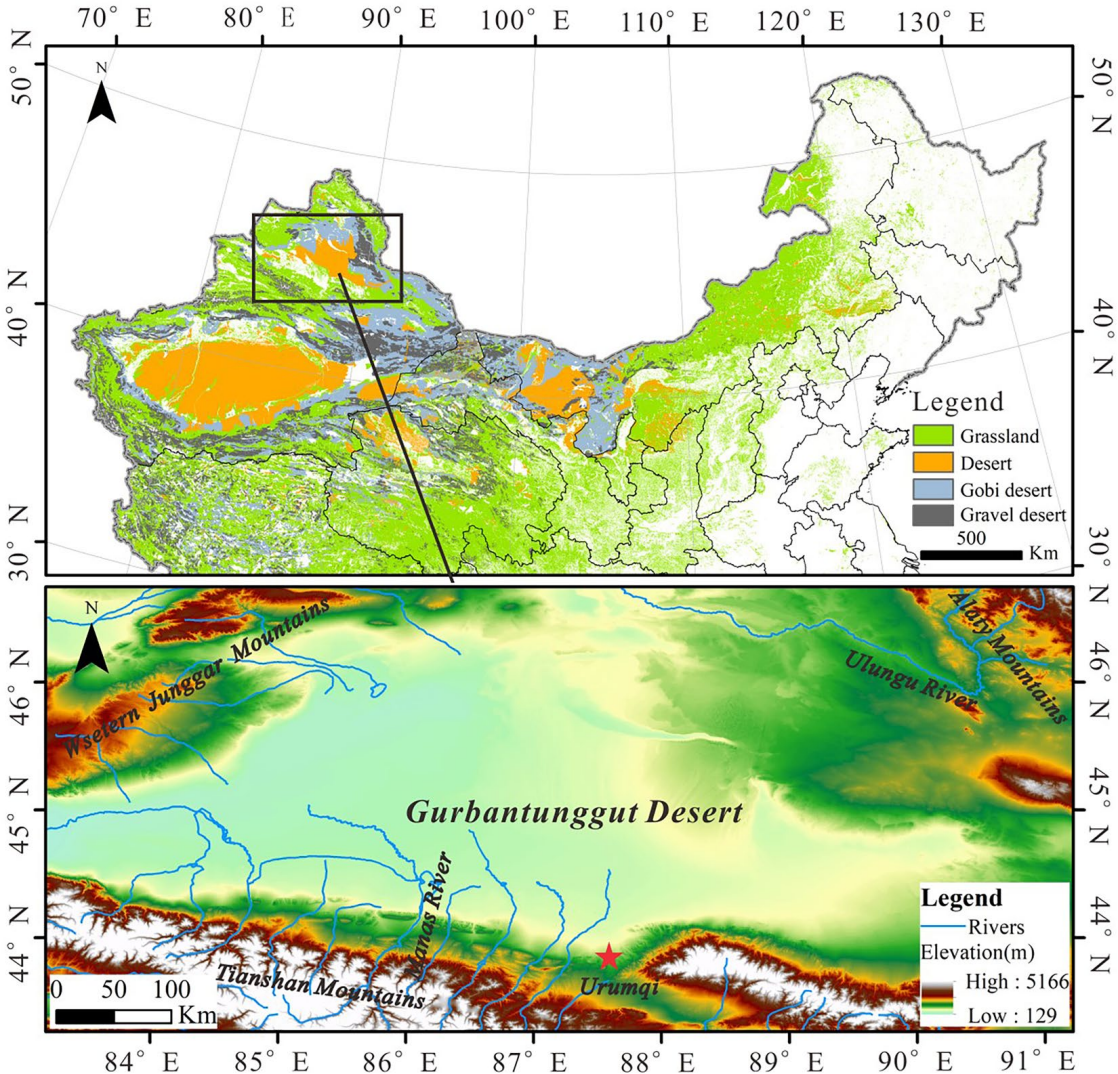
Geomorphologic background of the study area.

The Gurbantunggut Desert (44°11′–46°20′N, 84°31′–90°00′E) is situated in the interior of the Junggar Basin, Central Asia, bounded by the Tianshan Mountains, the Western Junggar Mountains, and the Altai Mountains. This vast alluvial–lacustrine plain hosts China’s second-largest desert, covering approximately 51,130 km², with an east–west extent of 480 km and a north–south width of about 230 km. It lies at an elevation of 450–600 m, making it the highest latitude desert in China and the only mid-latitude desert dominated by fixed and semi-fixed dunes^31^ (Fig. 1).

The region exhibits a typical temperate continental desert climate, with a mean annual temperature of 6.2 °C and annual precipitation ranging from 50 to 150 mm. The deeply buried groundwater and the orographic barrier effects of surrounding mountains block moisture influx and promote descending air currents, resulting in hot and arid summers. Conversely, its high latitude and proximity to the Siberian High cause cold winters with prolonged snow cover. Snowmelt during winter and spring infiltrates vertically, enhancing soil moisture and supporting extensive vegetation growth as temperatures rise.

Although the desert soils are nutrient-poor and low in fertility, abundant vegetation contributes significant organic matter that binds closely with soil particles, forming extensive biocrust layers. These biocrusts, up to 2 cm thick and widespread particularly in interdune areas, play a crucial role in sand stabilization and further promote vegetation establishment^32^. The overall vegetation cover ranges from 15–72%, with dominant species including *Haloxylon ammodendron*, *Anabasis aphylla*, *Ephedra distachya*, *Artemisia desertorum*, and *Ceratoides latens*. The desert also supports diverse fauna such as jerboas, hedgehogs, argali, and wild camels.

Under extensive vegetation and biocrust cover, most dune surfaces in the Gurbantunggut Desert remain largely stabilized, with minimal surface migration. However, dune crests, ridge tops, and upper slope positions are typically exposed and experience active sand transport driven by aeolian processes. Over longer time scales, these areas exhibit oscillatory dynamics and semi-fixed characteristics. Based on their dominant morphological features, the dunes can be classified into three major categories: transverse dunes (including barchan dunes, barchan dune chains, transverse ridges, and parabolic dunes); longitudinal dunes (linear ridges and dendritic dunes); and symmetrical dunes (checkerboard dune, honeycomb, and composite honeycomb forms). The distribution and morphology of these dune types are strongly controlled by regional wind regimes. Across a broad longitudinal span (~7°), prevailing arid airflow enters the desert from the west and northwest valleys, gradually transitioning into north-northwest (NNW) and northerly (N) winds. As a result, most dunes exhibit a dominant north–south orientation, especially dendritic dunes. Central basin convergence of abundant sand sources and intersecting winds facilitates the formation of honeycomb and complex Checkerboard dune patterns. In contrast, in the southeastern desert margin, wind directions shift to westerlies, sand supply diminishes, and dunes transition to a northwest–southeast (NW–SE) orientation, forming linear ridges and crescentic dunes.

## Sampling design and data acquisition

### Stage 1: Sample collection

To characterize sediment grain size variations among dune types, we first visually identified dune morphologies and spatial distributions using Google Earth imagery. Representative sampling areas were delineated and field sampling routes were planned accordingly. In total, sediment samples were collected from surface layers (0–1 cm)^33^, biocrust layers (0–2 cm)^34^, and vertical profiles (0–100 cm, or 0–60 cm in selected locations) in different dune morphological units.

Surface samples were obtained from ~20 cm × 20 cm flat sand surfaces using a stainless steel square shovel (262 mm length × 93 mm width × 47 mm height). Approximately 500 g of sand was collected per sample, excluding visible plant roots and animal feces, and stored in clean No. 8.5 sample bags. In biocrust-covered areas, a 0–2 cm thick layer (remove biocrust and root zone) was carefully bagged separately. For profile sampling, a pit 80–120 cm deep was excavated using a field shovel, and sediment was sampled at 10 cm intervals using 100 g aluminum boxes. All pits were subsequently backfilled to minimize environmental disturbance.

### Stage 2: Sample classification and spatial design

**Fig. 2:**
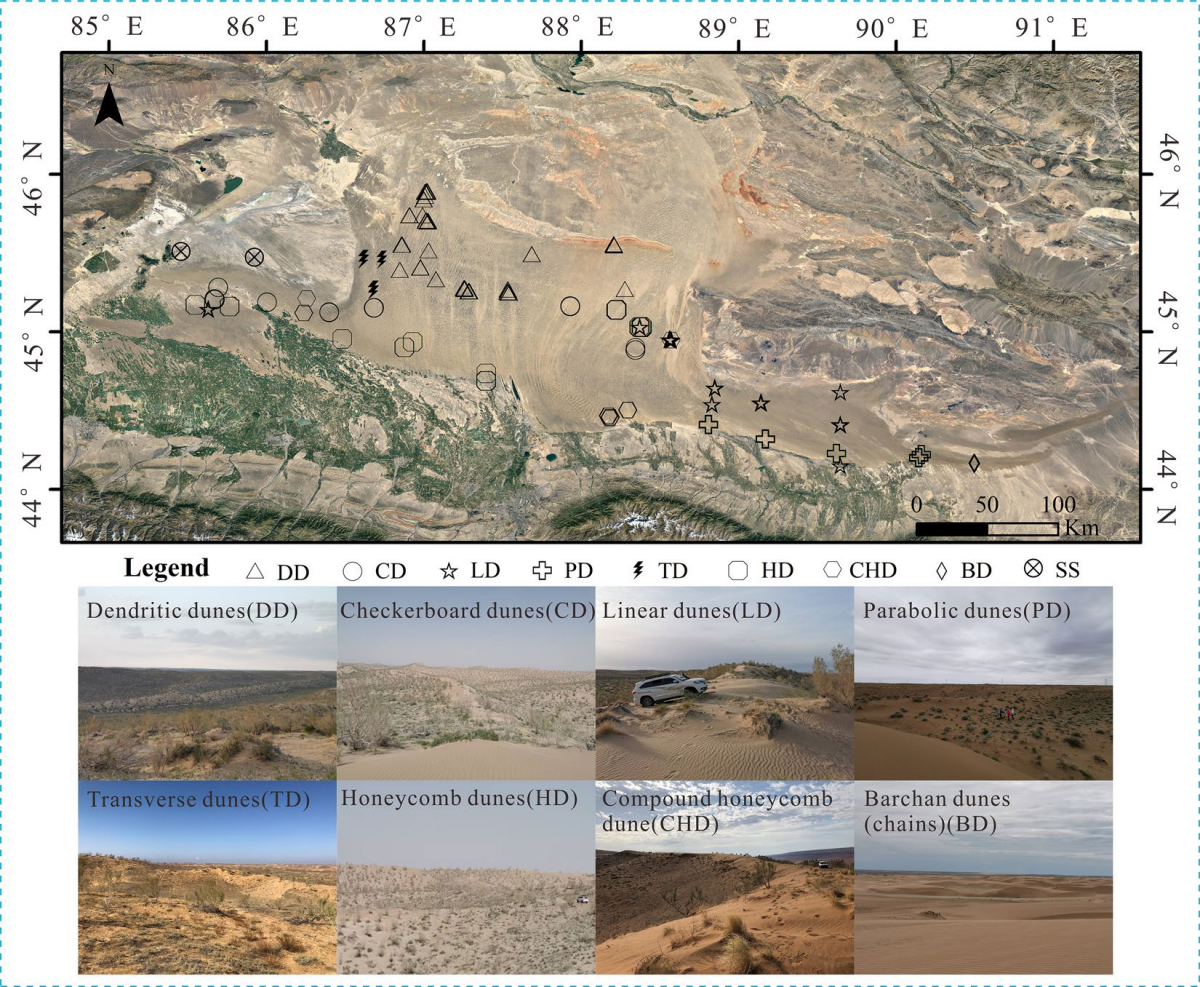
Geographic location of the study area and sampling points.

Sampling points were systematically positioned according to pre-defined geomorphological zoning and on-site conditions (Fig. 2). In total, 67 sampling zones and 424 sampling sites were established, resulting in 1,859 samples (bags or boxes). These zones comprehensively cover nearly all dune types and sand sheet (SS) in the Gurbantunggut Desert, excluding the easternmost star dunes.

For each typical dune, 6–7 sampling positions were selected, including dune crests, interdune surfaces, and multiple slope positions on both windward and leeward sides (summit, upper, middle, and base). Specifically, in crescent-shaped dunes (chains), samples were collected from interdune flats on the windward side, lower and middle windward slopes, dune crest, middle and base of leeward slopes, and interdune areas on the leeward side. In dendritic dunes and Y-shaped linear ridges, samples were obtained from slope bases, midslopes, crests, and intersections between primary and secondary ridges. For Checkerboard and honeycomb dunes, samples were taken from slope bases, midslopes, crests, and centers of Checkerboard or depression features. Linear ridges were sampled on both flanks, from slope base to crest. This sampling framework ensured comprehensive spatial coverage across diverse dune morphological units and geomorphic contexts.

## Data Record

The dataset, Comprehensive grain size database of sediments of different types of dunes in Gurbantunggut Desert^35^, has been uploaded to the Figshare and is available at 10.6084/m9.figshare.29596511. It consists of 3 data files, Excel DATA 1, DATA and DATA 3, describing the grain size characteristics of surface and profile sediments of different types of dunes in the Gurbantunggut Desert.

### DATA 1

Gurbantunggut Desert Dune Sediment Sample Database.

This document categorizes samples into nine groups based on dune types, containing the following Basic Information fields: (1) Sample number (in the third code field, a two-digit value denotes secondary ridges); (2) Geographic coordinates of sampling area; (3) Dune type; along with abbreviated names for nine dune types and their corresponding dune names; (4) Biocrust, biocrust presence at the surface is coded as “yes.”

### DATA 2

Database of sediment grain size characteristics for different types, locations, and depths in the Gurbantunggut Desert.

This database specifically includes the following data fields: (1) Sample number (which corresponds to data1 to indicate profile depth, in the third code field, each unit increment corresponds to a 10-cm depth interval); (2) Sediment grain size values. These raw measurement data can be effectively analyzed through various computational methods to evaluate sediment characteristics and their depositional environment.

### DATA 3

Particle Fraction Database of Sediments from the Gurbantunggut Desert.

This database consists of three sheet files, which are: (1) the grain size (μm) corresponding to the cumulative percentage of sediment; (2) particle size (ϕ) corresponding to converted sediment accumulation percentage using the particle size classification method proposed by Udden-Wentworth; and (3) four sediment particle size parameters calculated by the general formula proposed by Folk-Ward (MZ; $\sigma$, SK_g_; K_G_)^36^.

## Technical Validation

The grain size data of different types of dune sediments in the Gurbantunggut Desert underwent a rigorous validation process to ensure their accuracy, reliability and representativeness. A series of technical steps were carefully implemented throughout the data collection, processing and analysis phases to ensure the high quality and reproducibility of the results. Grain-size distributions were measured after pretreatment using a laser-diffraction particle-size analyzer (Mastersizer 2000, Malvern Instruments, UK; measurement range 0.02–2000 μm) at the Key Laboratory of the School of Geography and Tourism, Shaanxi Normal University.

### Pretreatment procedure

(1) Sediment samples were dry-sieved through a 10-mesh stainless-steel sieve (aperture ≈ 2.0 mm) to remove coarse debris, plant roots, and animal feces; (2) 5 g of sample was accurately weighed using an electronic balance (precision 0.001 g) and transferred into a beaker. Subsequently, 10 mL of 10% hydrogen peroxide (H₂O₂) was added to oxidize organic matter, and the mixture was heated on an electric hot plate at 200 °C until no visible bubbles were observed; (3) 10% hydrochloric acid (HCl) was then added to remove carbonates, again until no further bubbling occurred and the solution became clear; (4) the sample was rinsed repeatedly with distilled water and left to settle for at least 24 hours. After decanting the supernatant, 10 mL of 0.05 mol/L sodium hexametaphosphate [(NaPO₃)₆] solution was added as a dispersant, and the mixture was thoroughly shaken prior to measurement.

### Instrument operation

The analyzer was preheated for approximately 30 minutes. Operational settings included a stirrer speed of 800 rpm, pump speed of 2000 rpm, ultrasonic dispersion at 100%, and an obscuration range of 10–20%. The instrument was cleaned three times, and background values were measured before and between each sample run. Samples were sequentially introduced, and each measurement was performed after thorough cleaning and background calibration to ensure data accuracy.

### Data screening

To ensure accuracy, grain-size distributions were assessed for outliers and internal consistency. When replicate measurements of a sample still exhibited significant discrepancies after remeasurement, all data from the corresponding dune site were excluded. This conservative procedure ensured that the final dataset was free of systematic error and that all reported values were accurate and reproducible.

### Data interpretation and limitations

Surface samples were operationally defined as the uppermost 0–1 cm at bare-sand sites and 0–2 cm at biocrust-covered sites, consistent with field observations of biocrust thickness in the Gurbantunggut Desert. The dataset targets particle-size characteristics only; biocrust taxonomy, physiological status, and nutrient pools were not characterised.

## Data Availability

Sample Availability: All sediment samples included in this dataset have been preserved for potential future studies. Researchers wishing to access the physical samples or conduct additional analyses can request them directly from the authors, subject to academic use and logistical arrangements.

## Code availability

No specific code was used in the process of analyzing and calculating the comprehensive database of grain size characteristics of surface sediments of different types of dunes in the Gurbantunggut Desert.
